# Self-medication among Basic Science Medical Students of a Tertiary Care Centre: A Descriptive Cross-sectional Study

**DOI:** 10.31729/jnma.8497

**Published:** 2024-03-31

**Authors:** Prabineshwor Prasad Lekhak, Nischal Mainali, Swagata Mandal, Smriti Basnet, Natalia Oli

**Affiliations:** 1 Kathmandu Medical College and Teaching Hospital, Sinamangal, Kathmandu, Nepal; 2 Department of Community Medicine, Kathmandu Medical College and Teaching Hospital, Sinamangal, Kathmandu, Nepal

**Keywords:** *drugs*, *medical*, *prescription*, *self-medication*, *students*

## Abstract

**Introduction:**

Self-medication is using drugs to treat self-diagnosed signs or symptoms of oneself or others. Being closer to pharmacology and pathology, medical students have been more prone to it. The study aimed to determine the prevalence of self-medication among basic sciences medical students in a tertiary care centre in Kathmandu.

**Methods:**

A descriptive cross-sectional study was conducted among basic science students from 15 February 2023 to 14 March 2023 after obtaining ethical approval from the Institutional Review Committee (Reference number: 04122022/04). A convenience sampling method was used. Data were collected online and analysed. Point estimate at 95% confidence interval was calculated.

**Results:**

Among 322 basic science medical students the prevalence of self-medication was 218 (67.70%) (64.81-70.59 at 95% Confidence Interval). Fever was the most common condition treated by self-medication 106 (48.62%) and paracetamol was the most common medication 93 (42.66%). Of those who engaged in self-medication, 97 (44.50%) did so to save time. Moreover, within this group, 67 (30.73%) experienced adverse drug reactions, leading 37 (16.97%) of these students to visit a private doctor. Additionally, 138 (63.30%) refrained from prescribing medication to their family and friends.

**Conclusions:**

Self-medication among basic science medical students was found to be lower in comparison to other studies done in similar settings.

## INTRODUCTION

The World Health Organization (WHO) defines selfmedication as the "use of medicinal products by the consumer to treat self-recognized disorders or symptoms, or the intermittent or continued use of a medication prescribed by a physician for chronic or recurring diseases or symptoms. In practice, it also includes the use of the medication of family members, especially where the treatment of children or the elderly is involved."^1^

Self-medication reduces healthcare costs and enhances efficiency but poses risks due to potential misdiagnosis, incorrectmedicationchoices,dosages,or administration routes.^2^ Medical students are more likely to self-medicate due to their knowledge of pharmacology pathology and greater reach over drugs.^3^ Basic science students with limited clinical exposure face increased vulnerability to issues arising from incorrect medication choices, dosages, and associated side effects. Writing prescriptions for friends and family raises additional concerns about their self-medication practices.^4^

The study aimed to determine the prevalence of selfmedication among basic sciences medical students in a tertiary care centre.

## METHODS

This descriptive cross-sectional study was done among basic science medical students of Kathmandu Medical College Teaching Hospital, Duwakot, Bhaktapur, Nepal, after obtaining ethical approval from the Institutional Review Committee (Reference number: 04122022/04). The study was conducted from 15 February 2023 to 14 March 2023. In the study, basic sciences students i.e., students in their first year and second year of Bachelor of Medicine, Bachelor of Surgery (MBBS), Bachelor of Dental Surgery (BDS) and Bachelor of Physiotherapy (BPT) programs without clinical exposure were included. Nursing students have had clinical exposure since their initial years of study. Therefore, nursing students and students who denied consent were excluded from the study. A convenience sampling method was used due to practical constraints and limited access to the entire population. This approach was more feasible and cost-effective. The sample size was calculated by using the following formula:


$$\begin{array}{ll} \text{n} & ={\text{Z}}^{2}\times \frac{\text{p}\times \text{q}}{{\text{e}}^{\text{2}}}\\ & =1.96^{2}\times \frac{0.50\times 0.50}{0.05^{2}}\\ & =385 \end{array}$$

However, as basic science students' from MBBS, BDS, and BPT from the site of the study was 347, the sample size was calculated for a finite population which was 187.

After adding a 10% non-response rate, the sample size calculated was 200 medical Students. However, the final sample size collected was 322.

Data was collected using an online approach. The purpose of the study was explained to the respondents by sharing the easily understandable message through emails and social media groups. Emails were dispatched to their college email addresses, acquired through the college administration. Viber, Messenger, and WhatsApp groups of individual classes were also used to circulate forms for data collection. Moreover, they were also physically visited in groups and explained about the research and the time required to complete it. Follow-up was done to get responses. Confidentiality and anonymity were maintained. Participants in the study filled out a questionnaire on Google Forms and submitted it digitally after providing their digital consent.

A structured questionnaire was prepared using relevant literature. Similar questionnaire was already used in similar research conducted among undergraduate medical students.^5^ Additionally, a thorough literature review was conducted, and basic science specialists, pharmacologists, and subject matter experts were consulted to confirm the clarity and relevance of every question.

It contained three sections:

1. Socio-demographic characteristics and the participants' academic background, such as age, gender, faculty, and semester of study.
2. Use of self-medications during the last three months.
3. Those who practise self-medication filled in the remaining section. It included disease or symptom self-medicated for, class of drug used, reason for self-medication, the reason for self-medication, source for obtaining medication used, events of adverse drug reaction following self-medication, and aftermath of experiencing adverse drug reaction. Moreover, it included an attitude toward self-medication.

The collected data was analysed using Microsoft Excel 2018 and then analysed. Point estimate was calculated at 95% CI.

## RESULTS

Out of 322 basic science medical students, the prevalence of self-medication in the last three months was 218 (67.70%) (64.81-70.59 at 95% CI). From the age group 20-22 years there were 134 (61.46%) students. There were 132 (60.55%) males, and 86 (39.44%) females. It was seen that 126 (57.80%) MBBS students were involved in self medication (Table 1).

**Table 1:** Prevalence of self-medication according to the mentioned groups (n = 218).

Variables	n (%)
**Gender**	
Male	132 (60.55)
Female	86 (39.44)
**Faculty**	
MBBS	126 (57.80)
BDS	67 (30.73)
BPT	25 (11.47)
**Semester**	
First semester	46 (21.10)
Second semester	53 (24.31)
Third semester	54 (24.77)
Fourth semester	65 (29.82)

Amongst the total 106 (48.62%) used self-medication for fever while 101 (46.33%) for headache, 56 (25.69%) for cough and 55(25.23%) for body pain (Table 2).

**Table 2:** Conditions for self-medication (n = 218).

Variables	n (%)
Fever	106 (48.62)
Headache	101 (46.33)
Cough	56 (25.69)
Body pain	55 (25.23)
Running nose	54 (24.77)
Acidity	50 (22.94)
Diarrhoea	28 (12.84)
Muscle pain	27 (12.39)
Menstrual problems	24 (11.01)
Pain in joints	23 (10.55)
Vomiting	22 (10.09)
Nausea	20 (9.17)
Wounds	17 (7.80)
Difficulty in swallowing	16 (7.34)
Migraine	11 (5.05)
Dandruff	8 (3.67)
Eye infection	8 (3.67)
Skin diseases in open areas	8 (3.67)
Dental pain	7 (3.21)
Rash	7 (3.21)
Hair fall	6 (2.75)
Mouth ulcer	6 (2.75)
Skin disease in a covered area	6 (2.75)
Dysentery	5 (2.29)
Urination problems	5 (2.29)
Acne	2 (0.92)
Constipation	2 (0.92)
Ear pain	2 (0.92)
Genital Infection	2 (0.92)
Hypertension	2 (0.92)
Anaemia	1 (0.46)
Back pain	1 (0.46)
Common cold	1 (0.46)
Dengue	1 (0.46)
Skin disease in a covered area	1 (0.46)
Stomach pain	1 (0.46)

Analgesic and antipyretics were used by 115 (52.75%), anti-allergic- 31 (14.22%) and proton pump inhibitors and H2 blockers- 14(6.42%). Amongst analgesic and antipyretics, paracetamol was used by 93 (42.66%) and among anti-allergic, levocetirizine was used by 20 (9.17) (Table 3).

**Table 3:** Distribution according to the class of drugs used (n = 218).

Variables	n (%)
Analgesics and antipyretics	115 (52.75)
Paracetamol	93 (42.66)
Ibuprofen	19 (8.72)
Aspirin	2 (0.92)
Propyphenazone	1 (0.46)
Anti-allergy	31 (14.22)
Levocetirizine	20 (9.17)
Montelukast	3 (1.38)
Fexofenadine	3 (1.38)
Chlorpheniramine	3 (1.38)
Promethazine	1 (0.46)
Olopatadine	1 (0.46)
Antibiotics	28 (12.84)
Azithromycin	12 (5.50)
Metronidazole	5 (2.29)
Amoxicillin + Clavulanic acid	2 (0.92)
Ofloxacin	2 (0.92)
Ceftriaxone	1 (0.46)
Ciprofloxacin	1 (0.46)
Chloramphenicol	1 (0.46)
Minocycline	1 (0.46)
Mupirocin	1 (0.46)
Fusidic Acid	1 (0.46)
Doxycycline	1 (0.46)
Proton pump inhibitors and H2 blockers	14 (6.42)
Pantoprazole	12 (5.50)
Esomeprazole	1 (0.46)
Ranitidine	1 (0.46)
NSAIDs	7 (3.21)
Diclofenac	3 (1.38)
Nimesulide	2 (0.92)
Naproxen	1 (0.46)
Indomethacin	1 (0.46)
Antiemetic	5 (2.29)
Ondansetron	4 (1.83)
Domperidone	1 (0.46)
Antacid	4 (1.83)
Aluminium hydroxide + Magnesium hydroxide	3 (1.38)
Sodium bicarbonate + Citric acid + Sodium carbonate	1 (0.46)
**Miscellaneous**	
Decongestant	13 (5.96)
Antifungal	8 (3.67)
Vitamins & supplements	7 (3.21)
Ayurvedic	6 (2.75)
Opiod (Codeine)	3 (1.38)
Antitussive	3 (1.38)
Antimigraine	2 (0.92)
Local anaesthetic	2 (0.92)
Mucolytics	2 (0.92)
Laxative drugs	2 (0.92)
Corticosteroids	2 (0.92)
ORS	2 (0.92)
Bronchodilator	1 (0.46)
Antidiarrheal (loperamide)	1 (0.46)
Antidiabetic	1 (0.46)
Antidepressants	1 (0.46)
Antianxiety drugs	1 (0.46)

The reason for self-medication was the desire to save time, with 97 (44.50%) respondents indicating this as their primary motivation. The other reasons were using an old prescription 70 (32.11%), seeking pharmacist advice 41 (18.81%), the doctor being far away 27 (12.39%), high doctor fees 16 (7.34%), taking medicines prescribed for family or friends accounted for 16 (7.34%), and doctors being busy with many patients was 4 (1.83%).

When selecting a drug, among respondents who practised self-medication, 113 (51.83%) considered the brand when receiving the medicine, while 99 (45.41%) considered the pharmaceutical company. Price was regarded by 40 (18.35%) respondents, indicating its importance. Among students who practised self-medication, 150 (68.81%) did not experience adverse drug reactions, while 68 (31.19%) reported experiencing such reactions. After experiencing adverse drug reactions, 37 (16.97%) sought help from a private doctor, followed by 35(16.06%) who discontinued their medication and 12 (5.50%) visited the pharmacy. Only 7 (3.21%) respondents visited a primary health care centre in response to adverse drug reactions. Among students who practised self-medication, 173 (79.36%) identified the drugs they used. On the other hand, 33 (15.14%) respondents could not recognize the drugs they took, while 12 (5.50%) were uncertain about their identification.

About 138 (63.30%) respondents did not prescribe medication to their family and friends, while 80 (36.70%) did engage in such practices. Additionally, 181 (56.2%) respondents considered self-medication as a part of self-care. However, 235 (73.0%) respondents did not perceive self-medication as recommended by the WHO. Furthermore, 118 (36.6%) respondents believed that self-medication should be encouraged among medical students, whereas 237 (73.6%) would not recommend self-medication to others.

## DISCUSSION

The prevalence of self-medication among basic science students was 218 (67.70%). Previous studies conducted at the College of Medicine, NAIHS in Kathmandu, reported a prevalence of 76.6%.^4^ Similarly, self-medication was prevalent among undergraduate students at Lumbini Medical College at a rate of 83.3%.^6^ International studies conducted in countries such as India also highlighted the widespread practice of self-medication among medical students, with (92%) prevalence.^7^ Our study's prevalence rate is lower than all of these studies. This difference may be attributed to our study focusing specifically on basic science medical students, while the previous study included all medical students. Additionally, variations in sampling techniques, drug availability, and regulations regarding over-the-counter (OTC) medications may have influenced the prevalence rates.

The prevalence of self-medication was higher among MBBS students which is 126 (57.80%) when compared with other faculties. Our study found the highest prevalence of self-medication among fourth semester students which was similar to other studies, which revealed that self-medication increased each semester as students gained confidence in their knowledge of pharmacology, disease conditions, and prognosis.^8^

Fever was the most common illness for which selfmedication was practiced by 106 (48.62%) respondents which aligns with findings from another study conducted among second year (third and fourth semester) basic science students.^9^ Analgesics and antipyretics were the most commonly used class of drugs for selfmedication by 115 (52.75%) respondents, followed by anti-allergic by 31 (14.22%) and antibiotics by 28 (12.84%). Paracetamol emerged as the most frequently used drug for self-medication in the last three months by 93 (42.66%) respondents. These findings are also consistent with the study by Patil S. and Nagaiah B, where the analgesic-antipyretic class of drugs accounted for the majority of self-medication cases.^9^

In our study, the most common reason for selfmedication was a desire to save time according to 97 (44.50%) respondents. This finding differs from a study by Kumar et al., where illness deemed too trivial for consultation, was the most common reason for self-medication as responded by (70.5%) medical students.^10^ This discrepancy may be due to the time constraints students face as they dedicate most of their time to medical studies.^11^

Moreover, among all the respondents who engaged in self-medication in the last three months, the primary consideration when obtaining drugs was the brand by 113 (51.83%), followed by pharmaceutical companies by 99 (45.41%) and price by 40 (18.35%) respondents. This finding differs from other studies where, 40.7% considered anything pharmacists suggested, followed by (32.1%) who were concerned about side effects.^12^ The influence of marketing strategies employed by pharmaceutical companies, such as appealing brand names and packaging, likely contributes to the emphasis placed by medical students in our research on the brand and pharmaceutical companies when engaging in self-medication.^13^

A noteworthy 68 (31.19%) of the respondents who practicedself-medicationreportedexperiencingadverse drug reactions. This percentage is higher than in a similar study conducted in India, which reported adverse drug reactions in 8.25% of students.^14^ This difference may be attributed to variations in the types of medications used, as certain medicines have a higher association with adverse drug reactions. Variations in drug dosage and duration of use may be a factor in these variable rates.

Among those who experienced adverse drug reactions, 37 (16.97%) sought medical assistance from private doctors, followed by discontinuing their prescribed medications by 35 (16.06%) and visiting a pharmacy by 12 (5.50%) medical students. These findings are similar to a study conducted in a private institute in India, which reported that a significant proportion of individuals took some action following an adverse drug reaction, such as consulting a doctor or ceasing medication.^15^

It is worth noting that most respondents did not prescribe medication to family and friends. While most participants acknowledged that the World Health Organization does not recommend self-medication for others, they believed it was a part of self-care.

Although this research was carefully conducted, its limitations and shortcomings must be remembered. The study was done in one medical college in Kathmandu due to limited time and resources. So, the generalisation of study results cannot be made. The questionnaire used in the study was modified from a previous source, and validity and reliability testing were not conducted in the current setting.

## CONCLUSIONS

Self-medication among basic science medical students was found to be lower than in other studies done in similar settings. In this study, students commonly self-medicate for fever, headache, cough and bodyache. Analgesics and antipyretics were the most commonly used medicine.
